# A rapid and reliable method for early *Legionella pneumophila* identification and characterization in support of the epidemiology study

**DOI:** 10.3389/fmicb.2024.1452861

**Published:** 2024-10-08

**Authors:** Valentina Monistero, Nadia Vicari, Paola Prati, Roldano Bragoni, Alessandra Gazzola, Lorenza Sala, Antonio Maisano, Paolo Moroni, Valerio Bronzo, Mario Vittorio Luini, Bianca Castiglioni, Paola Cremonesi

**Affiliations:** ^1^Department of Veterinary Medicine and Animal Sciences - DIVAS, University of Milan, Lodi, Italy; ^2^Laboratorio di Malattie Infettive degli Animali - MiLab, University of Milan, Lodi, Italy; ^3^Diagnostic Section of Pavia, Istituto Zooprofilattico Sperimentale della Lombardia e dell’Emilia-Romagna - IZSLER, Pavia, Italy; ^4^Diagnostic Section of Lodi, Istituto Zooprofilattico Sperimentale della Lombardia e dell’Emilia-Romagna - IZSLER, Lodi, Italy; ^5^Institute of Agricultural Biology and Biotechnology – IBBA-CNR, National Research Council, Lodi, Italy

**Keywords:** Legionnaires’ disease, serogroup, virulence factor, TaqMan quantitative PCR, multiplex PCR serotyping, *Legionella pneumophila*, antimicrobial resistance

## Abstract

**Introduction:**

Legionnaires’ disease is a severe pneumonia predominantly caused by *Legionella pneumophila* (Lp), whose major reservoirs are artificial water systems. As most human infections are caused by *L. pneumophila* serogroup 1 (Lp1), a reliable method for Lp distinction can be crucial for bacterial spread prevention. As the ability to withstand in environments and to cause the waterborne disease is strongly related to specific genes, the identification of virulent strains can be of great relevance to implement water environmental monitoring and to contain harmful outbreaks to public health. We aimed to test an assay for Lp identification among different *Legionella* species, and to determine the serogroups. Additionally, we investigated the carriage of virulence and antimicrobial resistance genes.

**Methods:**

A total of 90 *Legionella* spp. isolates identified by phenotypic tests were subjected to the designed quantitative PCR assay targeting specific *mip* for Lp, *wzm* for Lp1, *pvcA* and *ahpD* for biofilm production. Eleven serogroups were investigated in all our isolates tested positive for *mip* gene, subsequently analyzed for 12 virulence and 8 antimicrobial resistance genes.

**Results:**

Only the 70 Lp isolates were positive for *mip*. Out of 27 Lp isolates belonging to serogroup 1 based on agglutination test, 23 (85.2%) carried *wzm*. The presence of *ahpD* and *pvcA* was found in 94.3 and 98.6% of Lp isolates, respectively. By multiplex PCR, all 23 *wzm*-positive strains were confirmed as serogroup 1 that was the most predominant (33%). At least one virulence gene was detected in all Lp isolates. The most frequent gene was *ispE* (100%), followed by *issD* (96%), *icmK* and *enhC* (93%), *cpxA* (91%), *rtxA2* (74%), *lvhB8-B9* (61%), and *prpA* (54%). The other genes were less diffused in Lp strains (*rtxA1,* 44%; *lvhB3-B4*, 47%; *pvcB*, 27%; *lvrE*, 24%). Of the macrolide resistance genes, the *ereA* was found in 84% of Lp strains, while only 14 (20%) harbored the *lpeAB* among the efflux pump genes.

**Conclusion:**

The assays validated in this study enable the simultaneous Lp and Lp1 detection. The differentiation of Lp strains according to their virulence properties could be useful to predict the bacterial ability to survive and to cause the disease.

## 1 Introduction

The genus *Legionella* includes 66 different species of intracellular Gram-negative bacteria (Parte et al., 2020), categorized into 80 distinct serogroups (SGs) (Mondino et al., 2020). These inhabitants of aquatic environment were first recognized as the causative agents of legionellosis in 1976, when a severe bacterial pneumonia outbreak was reported in Pennsylvania (Fraser et al., 1977; McDade et al., 1977). As artificial water systems are the major reservoirs, Legionnaires’ disease (LD) is predominantly caused by aerosol pathogen transmission in sufficient quantity (Prussin et al., 2017; Sawczyn-Domańska, 2021; Fasciana et al., 2023). After invading the lungs, the inhaled bacteria start replicating in alveolar macrophages (Khweek and Amer, 2010). This common waterborne disease can result in a multi-system inflammatory syndrome with acute respiratory symptoms, with a mortality rate ranging from 5 to 30% in immunocompromised patients (Dominguez et al., 2009; Phin et al., 2014). Considering the high risk of nosocomial infection in hospitalized people, the presence of *Legionella* spp. in the hospital environment has become a significant public health concern (Farnham et al., 2014). In the recent years, LD has shown a rising trend in both Europe and United States of America (USA), with contamination level of health care facilities higher than 79% in Italy (Napoli et al., 2010); most (90%) of clinical cases have been linked to *L. pneumophila* (Lp) (Miyashita et al., 2020). Although this species comprises at least 15 SGs, the majority of human infections are caused by SG1 (Kozak-Muiznieks et al., 2014). Due to its ability to survive for long periods in aquatic systems, Lp occurrence in hospital water samples should be periodically monitored by using rapid and reliable methods for the sensitive detection and the specific characterization of the isolates. For this purpose, a highly efficient multiplex PCR (mPCR) assay is available to quickly identify SGs, avoiding the use of typing sera and its poor reproducibility (Nakaue et al., 2021). For further epidemiological investigation, genotyping analysis can be applied to study the genetic diversity within Lp population (Sreenath et al., 2020). As significant differences exist among isolates belonging to the same serotype, molecular characterization can promote the understanding of Lp virulence (Qin et al., 2022). The distribution of genes involved in host cell attachment and replication and cell-to-cell spread, and their association with Lp pathogenesis can provide information on outbreak prediction and targeted treatment. Among the virulence factors, the macrophage infectivity potentiator encoded by *mip* is known to be highly specific for Lp (Engleberg et al., 1989; Sreenath et al., 2020). Furthermore, the genes encoding the *Legionella* vir homolog (*lvh*), essential for the survival in the environment, and the repeats in structural toxin (*rtxA*), a pore-forming toxin contributing to cellular entry and intracellular survival (D’Auria et al., 2008), have been frequently seen in Lp strains associated with human infections (Sawczyn-Domańska, 2021). The presence or absence of the cpxA protein, of the periplasmic protein EnhC required for bacterial replication within macrophages, and of the phage repressor encoded by *prpA* and enhancing immunoglobulin production and macrophage infection, have had significant effects on the ability to develop infection (Sawczyn-Domańska, 2021; Qin et al., 2022). Reliable monitoring of Lp virulence is important to control and cure LD appropriately. Antimicrobial therapy is usually based on active substances able to reach high intracellular concentrations, such as macrolides, fluoroquinolones, and rifampicin; especially azithromycin is recommended as first-line drug (Pedro-Botet et al., 2006). Despite the absence of resistance to these commonly used agents in Lp isolates, treatment failures were previously linked to the reduced azithromycin susceptibility due to the presence of *mefA/E* or *lpeAB* (Massip et al., 2017; Vandewalle-Capo et al., 2017). These genes were shown to encode components of a tripartite macrolide-specific efflux pump especially in *L. pneumophila* serogroup 1 (Lp1) (Jia et al., 2019), highlighting the need to control the emergence of resistant strains to clinically useful antibiotics.

As Lp represents a potentially serious threat to public health, we aimed to provide a rapid and reliable tool for its detection and characterization, and a further knowledge about the epidemiology. The aim of this study was to identify Lp among different *Legionella* species and to determine serogroups timely and accurately. The molecular characterization of the environmental isolates could help us to monitor Lp virulence and antimicrobial resistance, to investigate their diversity and their infection potential.

## 2 Materials and methods

### 2.1 Bacterial isolate collections and culture conditions

A total of 90 *Legionella* spp. strains were included in this study. Eighteen of them had been previously characterized by phenotypic and antigenic analysis, and then stored at the Territorial Section of Pavia of the Istituto Zooprofilattico Sperimentale della Lombardia e dell’Emilia Romagna (IZSLER). Two were obtained from the American Type Culture Collection (ATCC) and 16 from IZSLER collection as detailed in Table 1. The remaining 72 strains were instead isolated from water samples committed to the Territorial Section of Pavia of IZSLER from June 2022 to March 2024, during the routine activities. These water samples were aseptically collected from North Italian (Lombardia and Emilia Romagna regions) water systems, belonging to commercial activities (*n* = 23), hospitals (*n* = 12), private houses (*n* = 11), retirement homes (*n* = 6), dental offices (*n* = 6), hotels (*n* = 5), sports and recreation centers (*n* = 3), universities (*n* = 1), and other public facilities (*n* = 5). The 37 samples from Lombardia were collected in the provinces of Pavia (*n* = 17), Milano (*n* = 8), and Sondrio (*n* = 12), whereas the 35 samples from Emilia Romagna were collected in Ferrara (*n* = 2), Piacenza (*n* = 17), Ravenna (*n* = 8), and Modena (*n* = 8). All of them were immediately refrigerated, kept at 4°C and shipped to the Territorial Section of Pavia of IZSLER for bacterial isolation and identification, performed following the procedure described in UNI EN ISO 11731:2017 (International Organization for Standardization, 2017). All 90 *Legionella* spp. isolates from both collections and routine environmental monitoring were grown at 37°C on Buffered Charcoal Yeast Extract Agar (BCYE) and Glycine Vancomycin Polymyxin Cycloheximide Agar (GVPC) agar up to 10 days. Colonies were identified as *Legionella* spp. based on stereomicroscope observation of colony morphology on culture medium (BCYE and GVPC), absence of growth on blood agar, and agglutination on slide using polyclonal antisera. Lp1 and Lp serogroup 2–14 (Lp 2–14) were confirmed by a commercially available slide agglutination test (*Legionella* Latex Test-Thermo Scientific™ Oxoid, Basingstoke, United Kingdom).

**Table 1:** *Legionella* spp. strains from collections used as reference in this study.

Species	Strain	Serogroup
*Legionella anisa*	ATCC^1^ 35292	–
*Legionella bozemanii*	ATCC^1^ 33217	–
*Legionella micdadei*	IZSLER^2^ PV136464	–
*Legionella taurinensis*	IZSLER^2^ PV145431	–
*Legionella dumoffii*	IZSLER^2^ PV191339	–
*Legionella dumoffii*	IZSLER^2^ PV191418	–
*Legionella pneumophila*	IZSLER^2^ PV191413	2–14
*Legionella pneumophila*	IZSLER^2^ PV191453	2–14
*Legionella pneumophila*	IZSLER^2^ PV167249	2–14
*Legionella pneumophila*	IZSLER^2^ PV191444	2–14
*Legionella pneumophila*	IZSLER^2^ PV191445	2–14
*Legionella pneumophila*	IZSLER^2^ 23716/2023	2–14
*Legionella pneumophila*	IZSLER^2^ 409293/2022	1
*Legionella pneumophila*	IZSLER^2^ 162829/1–2022	1
*Legionella pneumophila*	IZSLER^2^ 23713/2023	1
*Legionella pneumophila*	IZSLER^2^ 23716b/2023	1
*Legionella pneumophila*	IZSLER^2^ 372960/2-2023	1
*Legionella pneumophila*	IZSLER^2^ 372960/4-2023	1

1 Reference strains obtained from the American Type Culture Collection.

2 Isolates from the Collection of Istituto Zooprofilattico Sperimentale della Lombardia e dell’Emilia-Romagna.

### 2.2 Genomic DNA extraction from pure cultures

DNA was isolated from each *Legionella* spp. strain (2–3 colonies from the same strain pure culture were suspended in PBS) using the commercial Mag Max™ CORE kit (Thermo Fisher Scientific, Rodano, Italy), following the manufacturer’s instructions.

### 2.3 TaqMan™ probe design for PCR amplification

Based on Lp genome sequences representing various serogroups available in the NCBI database,^1^ the *mip* gene was downloaded as species-specific target to discriminate Lp respect to *Legionella* spp., and the *wzm* gene to identify Lp1 (Collins et al., 2015). Similarly, *pvcA* and *ahpD* target genes were chosen based on sequences available in the same database to specifically detect Lp biofilm producing strains. After selection of the target genes, specific target probes were designed using Primer Express R v3.0 (Applied Biosystems, Foster City, CA, United States), by setting the annealing temperature of primers and probes at 60 and 70°C, respectively. The nucleotide BLAST tool^2^ was used to confirm the specificity of oligonucleotides *in silico*. Primers and TaqMan™ probes were synthesized by Applied Biosystems (Life Technologies Inc., Monza, Italy). Primers, 5′ 6-fluorescein-labeled (FAM) TaqMan probes, target genes, and reference sequences are listed in Table 2.

**Table 2:** TaqMan™ assays for qPCR.

Assay name	Target gene	Sequence	Accession number	Amplicon (bp)	Reference
Legpne-MIP	*mip*	GATTTGATGGCAAAGCGTACTG^1^ GGCTTCCCCTTTTACTTTATTTTCAT^2^ TGAATTCAATAAGAAAGCG^3^	MW053006.1	69	This study^4^
Legpne-WZM	*wzm*	TTTATGCCTCTGGCTTTGCA^1^ GGCACAGCAGAAACAGGGTAA^2^ TTATTTTATTACTCCACTCCAGCG^3^	HE980447.1	68	This study^4^
Legpne-AHPD	*ahpD*	CGCTTTGTGCATTTAACTGAAATT^1^ TTCCTTGCATTCGCAATCC^2^ AAATTGAGCATATTCCTGC^3^	LT632617.1	67	This study^4^
Legpne-PVCA	*pvcA*	GGGTCACCTGCCAGATCATG^1^ TGACAAAGTGTCCCCAAAAAATT^2^ TGAACGCCTTTCTCT^3^	AP024961.1	60	This study^4^

1 Primer forward.

2 Primer reverse.

3 Probe.

4 The assay used for the identification/characterization of Legionella was designed in this study.

### 2.4 TaqMan™ quantitative PCR assay for Lp detection

The DNAs from the 90 *Legionella* spp. (18 strains from ATCC and IZSLER collections, and 72 isolates from environmental samples) were tested to determine the analytical specificity of the designed qPCR assay for the presence of *mip* and *wzm* genes, and for the occurrence of the two genes involved in biofilm formation (*pvcA* and *ahpD*). The limit of detection (LoD) for each qPCR TaqMan assay was determined with strains from collections, starting from 40 ng/μL of the DNA template, using a 10-fold dilution, up to 0.04 pg/μL. Given that the genome of *Legionella* spp. is approximately 4.3 fg (Behets et al., 2006), the theoretical number of genomic DNA units (GU) of Lp in the extract was estimated to be around 1 × 10^7^ GU/μL. Subsequently, serial 10-fold dilutions in sterile water were performing, resulting in an external DNA standard ranging from 1 × 10^7^ to 1 × 10^1^ GU/μL. Reactions were carried out in 96-well plates sealed with adhesive optical covers (Applied Biosystems) and run on a QuantStudio™ 3 Real-Time PCR system (Applied Biosystems) for 2 min at 50°C, 10 min at 95°C, and 40 cycles of 15 s at 95°C and 1 min at 60°C. An identical thermal cycle was used for each target. All PCRs were done in duplicate. Each 20 μL of amplification reaction mix contained 1 μL of DNA (or ultrapure sterilized water for negative controls), 10 μL of TaqMan Environmental Master Mix 2.0 (2×), 1 μL of TaqMan assay 20× (18 μM for each primer, 5 μM for probe), 2 μL of TaqMan Exogenous Internal Positive Control (IPC) Reagents VICTM-labeled (ExoIPC Mix, Applied Biosystems), 0.4 μL of the Exo IPC DNA (target DNA) and 5.6 μL of molecular-grade water.

All the confirmed Lp collection strains and the identified Lp isolates from routine environmental monitoring were characterized by further molecular analysis.

### 2.5 Multiplex PCR serotyping

Firstly, 8 SGs (SG1, SG2, SG5, SG7, SG8, SG9, SG11, SG13) were investigated in all Lp isolates by a highly specific and sensitive mPCR assay, previously set up by Nakaue et al. (2021). Briefly, as previously described, the reaction mixtures were prepared in 0.2-mL tubes and performed in a 25-μL volume containing 0.2 μM of each primer set (from SG1 to SG13), 12.5 μL of Qiagen multiplex-PCR master mix (Qiagen, Hilden, Germany), and 1 μL of DNA (~140 ng/μL). Amplifications were carried out in a thermocycler (Biorad, Milan, Italy); a pre-PCR step was run at 94°C for 15 min followed by 30 PCR cycles under the following conditions: denaturation at 94°C for 1 min, annealing at 58°C for 1 min, and extension at 72°C for 1 min. The amplified PCR products were resolved using gel electrophoresis with 3% agarose gel (GellyPhor, Euroclone, Milan, Italy), stained with ethidium bromide (0.05 mg/mL; Sigma Aldrich, Milan, Italy) and visualized using a UV transilluminator (BioView Ltd., Nes Ziona, Israel). The DNA bands were compared with a molecular size marker (100-bp DNA ladder; Finnzymes, Espoo, Finland), and serogroups were determined following the sizes of amplification products, as previously described (Nakaue et al., 2021): SG1 = 249 base pairs (bp); SG2 = 543 bp; SG5 = 205 bp; SG7 = 835 bp; SG8 = 166 bp; SG9 = 634 bp; SG11 = 314 bp; SG13 = 461 bp. Subsequently, Lp isolates which did not generate any DNA amplicons were analyzed by standard PCR, using three different SG complexes (SG3/15, gene name *sg3-48/sg15/49*; SG6/12, gene name *sg12-57*; SG4/10/14 gene name *sg4-40/sg10-36/sg14-36* (*patA*)), and the same PCR conditions described above (Nakaue et al., 2021).

### 2.6 Standard PCR identification of virulence and antimicrobial resistance genes

All Lp isolates were subjected to the detection of 12 virulence genes (*pvcB*, *IssD, IspE, icmK, rtxA1, rtxA2, prpA, cpxA, enhC, lvhB3-B4, lvhB8-B9, lvrE*), and 8 antimicrobial resistance genes (*lpeAB, ereA, ereB, mefA, ermA, ermB, ermC, ermF*), using PCR analysis. The primer used in this study are reported in Table 3. All PCR reactions were carried out by using a thermocycler (Bio-Rad, Segrate, Italy), in 0.2-mL tubes containing 12.5 μL of 2× PCR Master Mix (Fermentas, M-Medical SRL, Milan, Italy), 0.2 μL of each of the primers, 1 μL of extracted DNA, and sterile water in a total reaction volume of 25 μL. For the target genes designed in this study, a pre-PCR step was run at 94°C for 5 min, followed by 30 PCR cycles of denaturation at 94°C for 1 min, annealing at 56°C for 1 min and extension at 72°C for1 min, and the final step at 72°C for 10 min to complete the reaction. For the remaining genes, amplification conditions of the specific references were followed. The amplified PCR products were resolved using gel electrophoresis with 2% agarose gel (GellyPhor, Euroclone, Milan, Italy), stained with ethidium bromide (0.05 mg/mL; Sigma Aldrich, Milan, Italy), and visualized under UV transilluminator (BioView Ltd., Nes Ziona, Israel), by using the 100-bp DNA ladder as molecular size marker (Finnzymes, Espoo, Finland).

**Table 3:** Sequences of PCR primers used in this study for analyzing virulence and antimicrobial resistance genes.

Gene name	Sequence	Reference
Biofilm formation		
*pvcB-for* *pvcB-rev*	CTGGACAAGGAGGAAGGACT TCCGGTGAGTAAAGAGCGTT	This study^1^
Virulence factors		
*issD-for*	TCACGGATGAGAGAGTTGAATC	This study^1^
*issD-rev*	GCTTCAATTAATAAAGTATCATCAAGCG	
*ispE-for*	ACTCTGTCTTCATAAGTTTCAATGTG	This study^1^
*ispE-rev*	GCCAAAAGCAATGGAATTGTTG	
*icmK-for*	GCTGATCAATCAGATGATGCTC	This study^1^
*icmK-rev*	GTGATAATCTTATTACTGGTGGTG	
*rtxA1-for*	GATCCGCAAGTAGCGCTCAC	Qin et al. (2022)
*rtxA1-rev*	TGTAATGCTGGCATTAGGCG	
*rtxA2-for*	CTGATGCTGCTACGGAACAC	Qin et al. (2022)
*rtxA2-rev*	CCGCAGTCATTACACCTGCG	
*prpA-for*	GTTTTAATCCCCCAGCAAGC	Qin et al. (2022)
*prpA-rev*	AATATCCCTACTCATCCTCG	
*cpxA-for*	ACAACCAGCTCGAGAGGA	Qin et al. (2022)
*cpxA-rev*	GCCATCACTTGGGAGTTC	
*enhC-for*	AATGCTTTGTATGCCCTCGG	Sawczyn-Domańska (2021)
*enhC-rev*	CATATCAGCGCTTTGGCCATC	
*lvhB3-for*	GGCTAGGAGGTTCTTGTG	Sawczyn-Domańska (2021)
*lvhB4-rev*	ATTGGCCGAGATGTCCTT	
*lvhB8-for*	CCTCTACGCATTACAACGCC	Sawczyn-Domańska (2021)
*lvhB9-rev*	GTGGTGGTAAAGGGAATGCC	
*lvrE-for*	GGTCCAATGGGTCCAGCAGG	Qin et al. (2022)
*lvrE-rev*	AGTGGCTGATTCTGGAGTGG	
Antimicrobial resistance		
*IpeAB-for*	GTGATGATTGTCTTATTGGTGCGA	Jia et al. (2019)
*IpeAB-rev*	ATGGCGTTTAAGATGATGGTGATT	
*ereA-for*	ATGAGTGTCATTGTGGGTCG	Jia et al. (2019)
*ereA-rev*	ATGACATCCCCTACTACACG	
*ereB-for*	AGAAATGGAGGTTCATACTTACCA	Jia et al. (2019)
*ereB-rev*	CATATAATCATCACCAATGGCA	
*mefA/E-for*	AGTATCATTAATCACTAGTGC	Jia et al. (2019)
*mefA/E-rev*	TTCTTCTGGTACTAAAAGTGG	
*ermA-for*	CTTCGATAGTTTATTAATATTAGT	Jia et al. (2019)
*ermA-rev*	TCTAAAAAGCATGTAAAAGAA	
*ermB-for*	AGTAACGGTACTTAAATTGTTTAC	Jia et al. (2019)
*ermB-rev*	GAAAAGGTACTCAACCAAATA	
*ermC-for*	GCTAATATTGTTTAAATCGTCAAT	Jia et al. (2019)
*ermC-rev*	TCAAAACATAATATAGATAAA	
*ermF-for*	CGGGTCAGCACTTTACTATTG	Jia et al. (2019)
*ermF-rev*	GGACCTACCTCATAGACAAG	

1 The assay used for the identification/characterization of Legionella was designed in this study.

### 2.7 Statistical analysis

Descriptive and inferential statistics were carried out using SPSS 29.0 (IBM, SPSS, Armonk, United States). The distribution of Lp1 and Lp 2–14 groups across regions was compared using a 2×2 contingency table and a Fisher exact test, while their frequencies at different sampling points categories, and the frequencies of virulence factors and antimicrobial resistance genes were compared with a binary logistic regression.

## 3 Results and discussion

### 3.1 *Legionella* speciation and Lp1 identification

Out of 90 *Legionella* spp. isolates (2 from ATCC collection, 16 from IZSLER collection, and 72 from environmental samples), we obtained a total of 70 Lp isolates (12 from IZLER collection and 58 from environmental samples) from positive colonies. The serogroups of all Lp strains were confirmed or determined via slide agglutination test, with 27 (39%) identified as Lp1 (6 from IZSLER collection and 21 from environmental samples). The remaining 43 (61%) isolates (6 from IZSLER collection and 37 from environmental samples) were classified as Lp 2–14. The statistical analysis revealed a different distribution of the two groups (Lp1 and Lp 2–14) across regions, with a significant association between Lp 2–14 and the environmental isolates from Lombardia (*p* = 0.002). Further results of Lp serogrouping, obtained by mPCR analysis, are described in 3.4 section.

### 3.2 Probe.design, analytical specificity, and sensitivity

Each TaqMan^®^ assay was analyzed *in silico* using a BLAST search on GenBank (see text footnote 2), confirming that only the targeted sequences were identified with 100% query cover and maximum identity. The specificity of the MIP and WZM TaqMan^®^ assays was confirmed by testing against the 18 *Legionella* spp. strains from ATCC and IZSLER collections, as listed in Table 1. In particular, the MIP assay targeted the sequence of the Lp species-specific *mip* gene, while the WZM assay was exclusive for Lp1. Out of these 18 strains, the *mip* gene was identified only in the 12 Lp strains without generating false positive or negative results (*data not shown*). Also, none of the six non*-pneumophila Legionella* strains were tested positive for the *mip* sequence detected by this qPCR assay, demonstrating the unique specificity and efficiency of this tool in detecting Lp. Simultaneously, WZM TaqMan^®^ assay confirmed 5 strains as Lp1. This molecular method enabled the detection of high-risk Lp1, and the results were subsequently confirmed by mPCR serogrouping in section 3.4, to ensure accuracy. As previously reported (Collins et al., 2015), the specificity of these optimized assays offers the potential for a rapid and selective approach during epidemiological investigations.

The analytical sensitivity of all TaqMan assays tested in triplicate in this study was approximately 0.4 pg/μL (10^2^ GU/μL) of total DNA, with mean cycle threshold (C_T_) values from 32.4 ± 0.14 for MIP TaqMan assay to 37.7 ± 0.22 for PVCA TaqMan assay. The MIP TaqMan assay also showed good sensitivity at 0.04 (35.3 ± 0.24; 10^1^ GU/μL) and 0.004 pg/μL (38.6 ± 0.72; 1 GU/μL), similar to previously obtained results (Benitez and Winchell, 2016).

### 3.3 Comparison of culture and qPCR for Lp and Lp1 detection

Out of 90 *Legionella* spp. strains included in this study, 70 isolates were correctly identified as Lp by the *mip* gene qPCR. Of these, 12 strains were obtained from the IZSLER collection and 58 isolates from environmental samples tested positive by culture (Table 4). These strains were also subjected to the *wzm* gene identification, resulting in a total of 23 *wzm*-positive strains. Of the six strains from the IZSLER collection identified as belonging to serogroup 1 by slide agglutination test, five were confirmed as Lp1 by *wzm* gene qPCR, as previously described. Out of 21 isolates from environmental samples that initially agglutinated with the SG1 antiserum, 18 (85.7%) were identified as Lp1, while three were not confirmed as Lp1 by qPCR. The WZM TaqMan® assay confirmed 85.2% of the positive results obtained by culture (Table 4), and correctly identified none of the strains as other serogroups, in agreement with previous studies (Mérault et al., 2011). In order to clarify the discrepancies between the two methods, the 4 strains not confirmed as Lp1 by qPCR were further investigated by mPCR as described below.

**Table 4:** Comparison of the detection of *Legionella pneumophila* (Lp; *mip*) and serogroup 1 (Lp1; *wzm*) by qPCR relative to culture results.

Species	Isolate		Lp *mip*		Lp1	*wzm*	
	n		n	%	n	n	%
Non-*pneumophila Legionella*	ATCC and IZSLER collections	6	0	0	–	0	0
	Environment	14	0	0	–	0	0
	Total	20	0	0	–	0	0
*Legionella pneumophila*	IZSLER collection	12	12	100	6	5	83.3
	Environment	58	58	100	21	18	85.7
	Total	70	70	100	27	23	85.2

### 3.4 SGs identification

**Figure 1:**
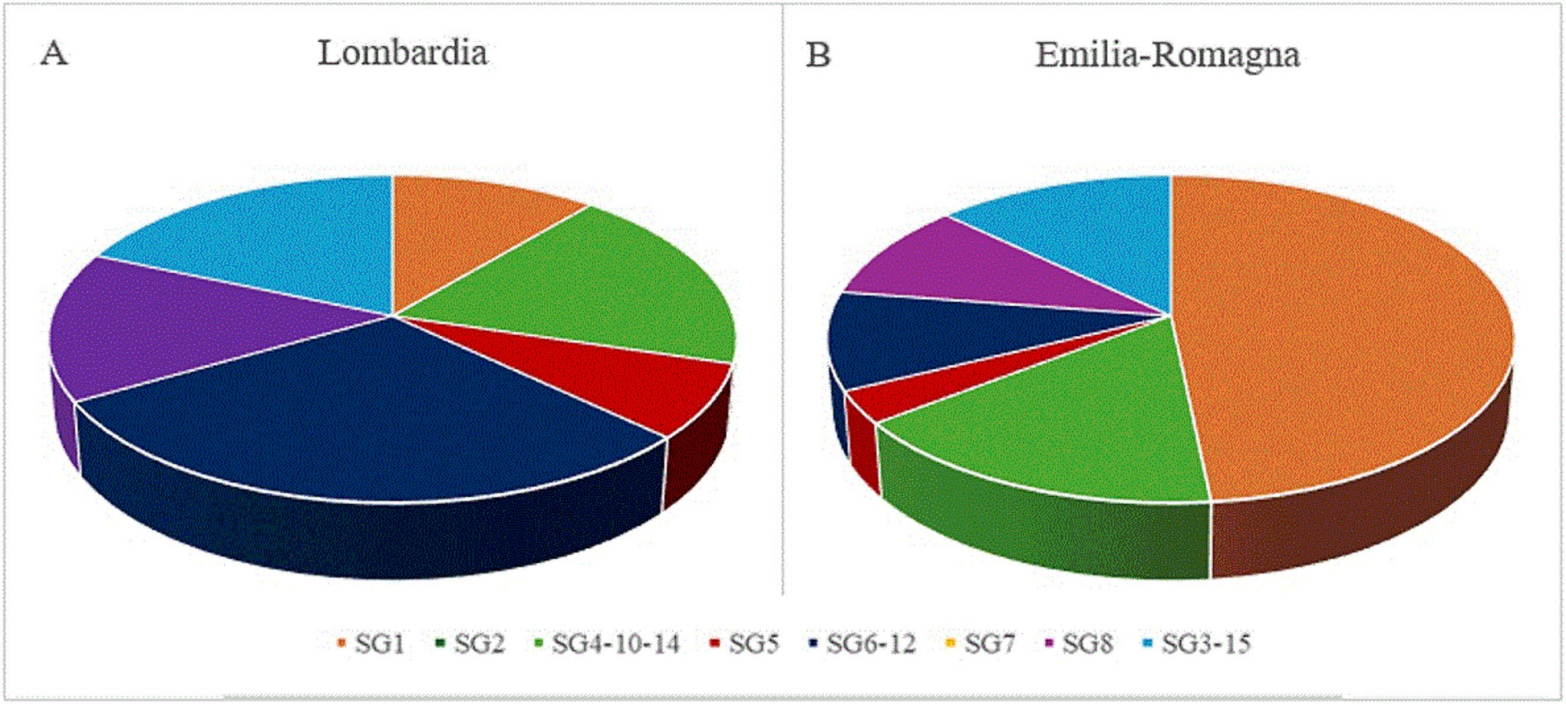
Distribution of serogroups (SG) in 58 *Legionella pneumophila* isolates from Lombardia **(A)** and Emilia-Romagna **(B)**, based on a mPCR serotyping assay.

The mPCR showed that 37 Lp isolates belonged to a single SG, while 33 to one of the three different SG complexes (3/15, 6/12 and 4/10/14). The PCR serotyping assay was not performed on the non*-pneumophila Legionella* isolates. Out of 70 Lp strains, the SG distribution in the 58 environmental isolates from different geographical regions is reported in Figure 1. The SG1 was recognized as the most predominant serogroup in Emilia-Romagna (Figure 1A; 48%), and its overall prevalence agreed with the results previously obtained in Japan (Komatsu et al., 2023). In the Southern Italy, on the contrary, positive samples with Lp of serogroup 2–15 were more frequent in different hospital areas, than SG1 (Arrigo et al., 2022). All 23 (33%) strains confirmed as SG1 using mPCR had been previously identified as Lp1 using agglutination test and, then, by qPCR. The results obtained for 4 *wzm*-negative strains were not consistent with those of the agglutination test, showing the higher accuracy of the *wzm* gene qPCR and validating the molecular support for culture-based detection in a frontline public health laboratory (Collins et al., 2015). In particular, one strain from the IZSLER collection previously belonging to serogroup 1 was classified into the SG complex 4/10/14, while three environmental Lp isolates showing agglutination in SG1 serum were grouped in SG4/10/14, SG6/12, and SG3/15, respectively. Facing the same Lp detection ratio, data in this study revealed a lower proportion of Lp1 results by qPCR compared to culture, as opposed to previous works (Collins et al., 2015). Being easier to interpret than culture, this sensitive and high throughput testing procedure could improve the rapid detection and the correct characterization of LD (Benitez and Winchell, 2013). Out of 70 Lp strains, the *wzm*-negative strains were classified mostly into the SG4/10/14 (19%; 13/70), followed by SG6/12 (16%; 11/70), and SG3/15 (13%; 9/70). Among these SG complexes, the SG6/12 (30%) was the most found in Lombardia, where also SG4/10/14 and SG3/15 were detected with higher frequency (19%) than in Emilia-Romagna (Figure 1B). Our findings were in agreement with previous outcomes in an Italian prison, where SG6 was the most prevalent serogroup, followed SG1 (Fasciana et al., 2019). These results confirmed Lp 2–14 as significantly prevalent in Lombardia. The statistically different distribution of Lp1 and Lp 2–14 between the two investigated regions confirmed that the diverse LD prevalence was related to Italian geographic variability (Fasciana et al., 2023), and suggested that the genetic diversity of Lp isolates could be related to their geographical origin. However, further studies are needed to correlate these findings with clinical samples, in order to investigate a link between the virulence of environmental isolates with LD outcomes. Despite all isolates coming from human-made environments, the region of interest might be useful for identifying harmful strains, requiring separate control and treatment measures. Although our findings agreed with previous ones (Komatsu et al., 2023), some strains identified as SG4/10/14 might belong to other serotypes, as they were subjected to PCR using primers designed to detect this SG complex according to the ORFs shared by SG4, SG5, SG7, SG8, SG9, SG13, and SG14 (Nakaue et al., 2021). All the others belonged to a single serogroup among SG8 (10%; 7/70), SG5 (6%; 4/70), SG7 (3%; 2/70), SG2 (1%; 1/70), and none was grouped in SG9, SG11, or SG13. The overall predominance of LD cases caused by Lp strains belonging to serogroup 1 (Yu et al., 2002) can be due to the possibility to detect only Lp1, using available diagnostic tests. As most LD cases are identified using the antigenic urinary test (ECDC, 2023) validated only for Lp1, the prevalence of all other serogroups can be underestimated and underdiagnosed (WHO, 2007) In this scenario, our study can provide further useful information to understand the environmental prevalence of Lp and, thus, monitoring its health hazard in order to predict the risk of possible outbreaks and to set up more targeted treatment.

### 3.5 Lp molecular characterization

At least one virulence-associated gene was detected in all Lp isolates. By qPCR, the occurrence of *ahpD* and *pvcA* genes was 94.3% (66/70) and 98.6% (69/70), respectively. Both genes involved in biofilm formation were simultaneously found in all the strain from IZSLER collection and in 53 environmental isolates while the other five were negative for either one (Table 5). These results suggested that Lp1 as well as Lp 2–14 could be resistant to water disinfection processes and, thus, could be difficult to eradicate from the environment (Fasciana et al., 2023). By PCR analysis, the most frequently detected gene was *ispE* and it was found in all Lp strains (100%), followed by *issD* (96%; 67/70), *icmK* and *enhC* (93%; 65/70), *cpxA* (91%; 64/70), *rtxA2* (74%; 52/70), *lvhB8-B9* (61%; 43/70), and *prpA* (54%; 38/70). The other genes, *rtxA1* (44%; 31/70) related to host cell adherence and cellular entry, *lvhB3-B4* (47%; 30/70) and *lvrE* (24%; 17/70) involved in secretion system, *pvcB* (27%; 17/70) responsible for biofilm formation, were detected in less than half of Lp strains. No significant differences in virulence distribution were observed between Lp1 and Lp 2–14 for *ispE* and *enhC,* confirming their essential role not only for bacterial pathogenesis but also for environmental survival (Samrakandi et al., 2002; Sawczyn-Domańska, 2021). Furthermore, *issD* and *icmK* genes were found with less frequency only in SG3/15 (78 and 67%, respectively). Of the genes involved in adherence (Cirillo et al., 2001), only 67% of the strains belonged to SG3/15 was positive for the *rtxA2*, that was less diffused also in SG1 (43%) as well as *rtxA1* (52%), both of which were previously found more often in isolates from human infections (Sreenath et al., 2020). The less distributed *rtxA1* showed a stronger association with SG2 (100%), SG8 (86%), and SG4/10/14 (69%) than with SG5 (25%) and SG3/15 (22%); moreover, its presence was not detected in SG7 and SG6/12. Among the genes related to the secretion system (Buse et al., 2015), higher variability was found for those encoding *Legionella* vir homolog (*lvh*), and contributing to conjugation and virulence (Segal et al., 1999). Both *lvhB3-B4* and *lvhB8-B9* genes were carried by both strains of SG7 (100%), and more than half of those grouped in SG8 (71 and 57%, respectively), SG4/10/14 (62 and 77%, respectively), and SG 3/15 (56%). On the other hand, *lvhB3-B4* than *lvhB8-B9* were less linked to SG1 (35 and 52%, respectively), SG2 (0 and 100%, respectively), SG5 (0 and 50%, respectively), and SG6/12 (18 and 64%, respectively). The simultaneous absence of *lvh* and *rtxA* was not registered in any Lp strains, while both genes encoding the *Legionella* vir homolog (either *lvhB3-B4* or *lvhB8-B9* or both) and the *dot/icm*-regulated pore-forming toxin (either *rtxA1* or *rtxA2* or both) were detected in 51 (72.9%) of them. Most isolates bearing *rtxA* but not *lvh* belonged to SG1 (40%), followed by SG6/12 (26%) and SG5 (13%), in addition to one isolate of SG8 (7%), one of SG4/10/14 (7%), and one of SG 3/15 (7%). On the contrary, four isolates with *lvh* but without *rtxA*, two grouped in SG1 and two in SG6/12, may show a reduced ability for attachment, cytotoxicity, and intracellular growth (Cirillo et al., 2001). Although their role might not be essential to survive in aquatic environments, the statistically analysis showed the significant occurrence of *rtxA2* in Lp 2–14 isolates (*p* = 0.02), confirming that the association with *lvh* could be used as indicators of Lp infection potential (Huang et al., 2004; Huang et al., 2006). Moreover, the *lvrE* was statistically significant related with Lp1 (*p* = 0.035), whose presence together with *lvhB3-B4* or *lvhB8-B9* was found in 43% of SG1 and SG8 isolates other than three strains of SG4/10/14 and one of SG7. Among the genes involved in host cell adherence and cellular entry, the *cpxA* was widely spread, with the slight exception of SG5 (75%) and SG8 (67%), that could be more virulent precisely for this lack (Qin et al., 2022). On the contrary, greater variation was seen for *prpA,* found in both strains of SG7 (100%) and in most strains of SG8 (71%), SG3/15 (67%), and SG1(61%), as well as in around half of the strains belonging to SG4/10/14 and SG5, but only 18% of the strains grouped in SG6/12; the absence of this gene could be related to a significantly lower virulence in this SG complex (Qin et al., 2022). Even the gene responsible for biofilm production (*pvcB*) displayed high distribution diversity, with a frequency of 100% in SG2, 50% in SG5 and SG7, and 45% in SG6/12; only 33% of SG3/15, 23% of SG4/10/14 and 17% of SG1 isolates were positive for this gene, that was not detected in any strain grouped in SG8. We explored the presence of three different macrolide antibiotic resistance mechanisms, encoded by methylase genes (*ermA, ermB, ermC,* and *ermF*), macrolide esterase genes (*ereA* and *ereB*), and efflux pump genes (*mefA* and *lpeAB;* Massip et al., 2017). Their concurrent absence was observed in 11 (16%) Lp positive samples, most of which belonging to SG1. As shown in Table 6, the *ereA* was found in 84% of Lp strains, while none was positive for *ereB,* as well as for all *erm* genes (*ermA, ermB, ermC* and *ermF*). Macrolides and fluoroquinolones are still the most used therapeutic agents to treat LD, as exert a bacteriostatic effect by interacting with the site of peptide bond formation and inhibiting protein synthesis disease (Pedro-Botet et al., 2006; Woodhead et al., 2011). However, the high frequency of *ereA-*positive strains detected in Italy compared to China, where both macrolide esterase (*ereA* and *ereB*) and methylase (*erm*) genes were not previously observed (Jia et al., 2019), may suggest a decreased efficiency of this antimicrobial class to treat environmental isolates of Lp. On the other hand, the efflux pump genes (*mefA* and *lpeAB*) were investigated to explore the reduced azithromycin susceptibility and their presence agreed with previous data (Jia et al., 2019). The *mefA* was not detected in any isolate, while only 14 (20%) Lp isolates possessed the *lpeAB*. In particular, the only one (100%) of SG2 carried both *ereA* and *lpeAB,* while all (100%) strains grouped in SG7 and SG6/12 were positive for *ereA*, but negative for *IpeAB* (0%). This gene was not amplified also in SG3/15 and SG5, that harbored the other macrolide resistance gene (*ereA*) with a frequency of 89 and 75%, respectively. Interestingly, the highest prevalence of *lpeAB* (43 and 35%, respectively) was found in SG8 and SG1, bearing *ereA* in 71 and 78% of isolates, respectively.

**Table 5:** Detection of virulence genes in *Legionella pneumophila* (Lp) isolates grouped by serogroups.

Lp		Virulence factors																											
		Biofilm formation						Bacterial survival and intracellular replication								Host cell adhesion				MDR Regulation				Secretion system					
SG*	N	*ahpD*		*pvcA*		*pvcB*		*enhC*		*issD*		*ispE*		*icmK*		*rtxA1*		*rtxA2*		*prpA*		*cpxA*		*lvhB3-B4*		*lvhB8-B9*		*lvrE*	
		%	N	%	N	%	N	%	N	%	N	%	N	%	N	%	N	%	N	%	N	%	N	%	N	%	N	%	N
1	23	87	20	100	23	17	4	87	20	100	23	100	23	96	22	52	12	43	10	61	14	96	22	35	8	52	12	43	10
2	1	100	1	100	1	100	1	100	1	100	1	100	1	100	1	100	1	100	1	0	0	100	1	0	0	100	1	0	0
4/10/14	13	100	13	100	13	23	3	92	12	100	13	100	13	100	13	69	9	100	13	54	7	92	12	62	8	77	10	23	3
5	4	100	4	100	4	50	2	100	4	100	4	100	4	100	4	25	1	100	4	50	2	75	3	0	0	50	2	0	0
6/12	11	100	11	91	10	45	5	100	11	100	11	100	11	91	10	0	0	82	9	18	2	100	11	18	2	64	7	0	0
7	2	100	2	100	2	50	1	100	2	100	2	100	2	100	2	0	0	100	2	100	2	100	2	100	2	100	2	50	1
8	7	86	6	100	7	0	0	86	6	86	6	100	7	100	7	86	6	100	7	71	5	57	4	71	5	57	4	43	3
3/15	9	100	9	100	9	33	3	100	9	78	7	100	9	67	6	22	2	67	6	67	6	100	9	56	5	56	5	0	0
Total	70	94	66	99	69	27	19	93	65	96	67	100	70	93	65	44	31	74	52	54	38	91	64	43	30	61	43	24	17

*Serogroups as previously named by Nakaue et al., 2021.

**Table 6:** Detection of azithromycin resistance genes in *Legionella pneumophila* (Lp) isolates grouped by serogroups.

Lp		Azithromycin resistance			
SG	N	*lpeAB*		*ereA*	
		%	N	%	N
1	23	35	8	78	18
2	1	100	1	100	1
4/10/14	13	15	2	85	11
5	4	0	0	75	3
6/12	11	0	0	100	11
7	2	0	0	100	2
8	7	43	3	71	5
3/15	9	0	0	89	8
Total	70	20	14	84	59

## 4 Conclusion

As Lp continues to be a potentially serious threat to human health, especially due to the wide spread of Lp1, a fast and accurate monitoring technique can be crucial for an effective LD prevention and control. The assays validated in this study enable the simultaneous Lp detection and distinction. In routine laboratory testing of *Legionella* spp., the Lp identification at species level is required to subsequently define the specific serogroups by using slide agglutination test. However, the serogroup assignment of the isolates agglutinating to specific monoclonal antibodies is expensive and time consuming. Although bacterial culture remains the gold standard for the LD diagnosis, this study proposes a qPCR approach as a rapid and reliable supplementary tool for Lp culture-based distinguishing. Both Lp and Lp1 detection could be performed in one-step process, supporting a timely and cost-effective epidemiological investigation of LD outbreaks. The molecular approach can be used for surveillance purposes and can be applied to screen environmental samples. The synergic use of this molecular method in addition with the previously described routine diagnostic tests may help with more effective responses in public health protection. Although this assay was focused on Lp1, being the most frequently associated with human diseases, the same approach can be further applied to other serogroups. But meanwhile, the mPCR still represents an additional and suitable molecular technique for SG differentiation, increasing the opportunity to improve the diagnostic performance in classifying Lp isolates other than Lp1 and yielding a better understanding of the LD causative agent. As the results showed the genetic diversity even within Lp isolates being of same serotypes, the results can be further supplemented by *in vitro* macrophage assay, in order to correlate their virulence profiles with the bacterial survival ability and, thus, to predict the capacity to cause the disease. The molecular identification and characterization of virulent strains can be of great relevance to implement control measures of water environment and to prevent human cases. Although a subpopulation carried genes responsible for reducing macrolide and azithromycin susceptibility, most of the isolates analyzed in this study showed the absence of resistance genes to the most frequently used antimicrobial to treat LD.

## Data Availability

The raw data supporting the conclusions of this article will be made available by the authors, without undue reservation.

## Footnotes

^1^https://www.ncbi.nlm.nih.gov/pubmed/

^2^https://blast.ncbi.nlm.nih.gov/Blast.cgi

## References

[ref2] Arrigo I. Galia E. Fasciana T. Diquattro O. Tricoli M. R. Serra N.. (2022). Four-year environmental surveillance program of *Legionella* spp. in one of Palermo's largest hospitals. Microorganisms 10:764. doi: 10.3390/microorganisms10040764, PMID: 35456814 PMC9030258

[ref3] Behets J. Declerck P. Delaedt Y. Creemers B. Ollevier F. (2006). Development and evaluation of a Taqman duplex real-time PCR quantification method for reliable enumeration of *Legionella pneumophila* in water samples. J. Microb. Methods 68, 137–144. doi: 10.1016/j.mimet.2006.07.002, PMID: 16914218

[ref4] Benitez A. J. Winchell J. M. (2013). Clinical application of a multiplex real-time PCR assay for simultaneous detection of Legionella species, Legionella pneumophila, and *Legionella pneumophila* serogroup 1. J. Clin. Microbiol. 51, 348–351. doi: 10.1128/JCM.02510-12, PMID: 23135949 PMC3536254

[ref5] Benitez A. J. Winchell J. M. (2016). Rapid detection and typing of pathogenic nonpneumophila Legionella spp. isolates using a multiplex real-time PCR assay. Diagn. Microbiol. Infect. Dis. 84, 298–303. doi: 10.1016/j.diagmicrobio.2016.01.007, PMID: 26867966 PMC8972187

[ref6] Buse H. Y. Lu J. Ashbolt N. J. (2015). Exposure to synthetic gray water inhibits amoeba encystation and alters expression of *Legionella pneumophila* virulence genes. Appl. Environ. Microbiol. 81, 630–639. doi: 10.1128/AEM.03394-14, PMID: 25381242 PMC4277573

[ref7] Cirillo S. L. Bermudez L. E. El-Etr S. H. Duhamel G. E. Cirillo J. D. (2001). *Legionella pneumophila* entry gene rtxA is involved in virulence. Infect. Immun. 69, 508–517. doi: 10.1128/IAI.69.1.508-517.2001, PMID: 11119544 PMC97910

[ref8] Collins S. Jorgensen F. Willis C. Walker J. (2015). Real-time PCR to supplement gold-standard culture-based detection of Legionella in environmental samples. J. Appl. Microbiol. 119, 1158–1169. doi: 10.1111/jam.12911, PMID: 26218315

[ref9] D’Auria G. Jiménez N. Peris-Bondia F. Pelaz C. Latorre A. Moya A. (2008). Virulence factor RTX in *Legionella pneumophila*, evidence suggesting it is a modular multifunctional protein. BMC Genomics 9:14. doi: 10.1186/1471-2164-9-14, PMID: 18194518 PMC2257941

[ref10] Dominguez A. Alvarez J. Sabria M. Carmona G. Torner N. Oviedo M.. (2009). Factors influencing the case-fatality rate of Legionnaires’ disease. Int. J. Tuberc. Lung Dis. 13, 407–412, PMID: 19275805

[ref11] ECDC (2023). Legionnaires’ disease. Annual epidemiological report for 2021. Stockholm: European Centre for Disease Prevention and Control.

[ref16] Engleberg N. C. Carter C. Weber D. R. Cianciotto N. P. Eisenstein B. I. (1989). DNA sequence of mip, a *Legionella pneumophila* gene associated with macrophage infectivity. Infect. Immun. 57, 1263–1270. doi: 10.1128/iai.57.4.1263-1270.1989, PMID: 2925252 PMC313259

[ref12] Farnham A. Alleyne L. Cimini D. Balter S. (2014). Legionnaires’ disease incidence and risk factors, New York, New York, USA, 2002–2011. Emerg. Infect. Dis. 20, 1795–1802. doi: 10.3201/eid2011.131872, PMID: 25513657 PMC4214295

[ref13] Fasciana T. Mascarella C. Distefano S. A. Calà C. Capra G. Rampulla A.. (2019). Cluster of Legionnaires’ disease in an Italian prison. Int. J. Environ. Res. Public Health 16:2062. doi: 10.3390/ijerph16112062, PMID: 31212678 PMC6604178

[ref14] Fasciana T. Palermo M. Arrigo I. Tricoli M. R. Diquattro O. Giammanco A. (2023). Editorial: special issue: *Legionella pneumophila*: a microorganism with a thousand faces. Microorganisms 11:2392. doi: 10.3390/microorganisms11102392, PMID: 37894050 PMC10609420

[ref15] Fraser D. W. Tsai T. R. Orenstein W. Parkin W. E. Beecham H. J. Sharrar R. G.. (1977). Legionnaires’ disease: description of an epidemic of pneumonia. N. Engl. J. Med. 297, 1189–1197. doi: 10.1056/NEJM197712012972201 335244

[ref18] Huang B. Heron B. A. Gray B. R. Eglezos S. Bates J. R. Savill J. (2004). A predominant and virulent *Legionella pneumophila* serogroup 1 strain detected in isolates from patients and water in Queensland, Australia, by an amplified fragment length polymorphism protocol and virulence gene-based PCR assays. J. Clin. Microbiol. 42, 4164–4168. doi: 10.1128/JCM.42.9.4164-4168.2004, PMID: 15365006 PMC516327

[ref19] Huang B. Yuan Z. Heron B. A. Gray B. R. Eglezos S. Bates J. R.. (2006). Distribution of 19 major virulence genes in *Legionella pneumophila* serogroup 1 isolates from patients and water in Queensland, Australia. J. Med. Microbiol. 55, 993–997. doi: 10.1099/jmm.0.46310-0 16849718

[ref17] International Organization for Standardization (2017). ISO 11731:2017, water quality - enumeration of legionella. Available at: https://www.iso.org/standard

[ref20] Jia X. Ren H. Nie X. Li Y. Li J. Qin T. (2019). Antibiotic resistance and azithromycin resistance mechanism of *Legionella pneumophila* serogroup 1 in China. Antimicrob. Agents Chemother. 63, e00768–e00719. doi: 10.1128/AAC.00768-19, PMID: 31405864 PMC6761501

[ref21] Khweek A. A. Amer A. (2010). Replication of *Legionella pneumophila* in human cells: why are we susceptible? Front. Microbiol. 1:133. doi: 10.3389/fmicb.2010.00133 21687775 PMC3109522

[ref22] Komatsu S. Tanaka S. Nakanishi N. (2023). Evaluation of *Legionella pneumophila* SGUT serotypes isolated from Bath water using a multiplex-PCR serotyping assay. Jpn. J. Infect. Dis. 76, 77–79. doi: 10.7883/yoken.JJID.2022.397, PMID: 36047173

[ref23] Kozak-Muiznieks N. A. Lucas C. E. Brown E. Pondo T. Taylor T. H. Frace M.. (2014). Prevalence of sequence types among clinical and environmental isolates of *Legionella pneumophila* serogroup 1 in the United States from 1982 to 2012. J. Clin. Microbiol. 52, 201–211. doi: 10.1128/JCM.01973-13, PMID: 24197883 PMC3911437

[ref24] Massip C. Descours G. Ginevra C. Doublet P. Jarraud S. Gilbert C. (2017). Macrolide resistance in *Legionella pneumophila*: the role of LpeAB efflux pump. J. Antimicrob. Chemother. 72, 1327–1333. doi: 10.1093/jac/dkw594, PMID: 28137939

[ref25] McDade J. E. Shepard C. C. Fraser D. W. Tsai T. R. Redus M. A. Dowdle W. R. (1977). Legionnaires’ disease: isolation of a bacterium and demonstration of its role in other respiratory disease. N. Engl. J. Med. 297, 1197–1203. doi: 10.1056/NEJM197712012972202 335245

[ref26] Mérault N. Rusniok C. Jarraud S. Gomez-Valero L. Cazalet C. Marin M.. (2011). Specific real-time PCR for simultaneous detection and identification of *Legionella pneumophila* serogroup 1 in water and clinical samples. Appl. Environ. Microbiol. 77, 1708–1717. doi: 10.1128/AEM.02261-10, PMID: 21193672 PMC3067292

[ref27] Miyashita N. Higa F. Aoki Y. Kikuchi T. Seki M. Tateda K.. (2020). Distribution of Legionella species and serogroups in patients with culture-confirmed Legionella pneumonia. J. Infect. Chemother. 26, 411–417. doi: 10.1016/j.jiac.2019.12.016, PMID: 32081644

[ref28] Mondino S. Schmidt S. Rolando M. Escoll P. Gomez-Valero L. Buchrieser C. (2020). Legionnaires’ disease: state of the art knowledge of pathogenesis mechanisms of Legionella. Annu. Rev. Pathol. 15, 439–466. doi: 10.1146/annurev-pathmechdis-012419-032742 31657966

[ref29] Nakaue R. Qin T. Morita M. Ren H. Chang B. Murai M.. (2021). Development of a multiplex-PCR serotyping assay for characterizing *Legionella pneumophila* serogroups based on the diversity of lipopolysaccharide biosynthetic loci. J. Clin. Microbiol. 59:e0015721. doi: 10.1128/JCM.00157-21, PMID: 34379526 PMC8525581

[ref30] Napoli C. Fasano F. Iatta R. Barbuti G. Cuna T. Montagna M. T. (2010). Legionella spp. and legionellosis in southeastern Italy: disease epidemiology and environmental surveillance in community and health care facilities. BMC Public Health 10:660. doi: 10.1186/1471-2458-10-660, PMID: 21044294 PMC2988737

[ref31] Parte A. C. Sardà Carbasse J. Meier-Kolthoff J. P. Reimer L. C. Göker M. (2020). List of prokaryotic names with standing in nomenclature (LPSN) moves to the DSMZ. Int. J. Syst. Evol. Microbiol. 70, 5607–5612. doi: 10.1099/ijsem.0.004332, PMID: 32701423 PMC7723251

[ref32] Pedro-Botet M. L. Garcí-Cruz A. Tural C. Mateu L. Sopena N. Roure S. (2006). Severe Legionnaires’ disease successfully treated with levofloxacin and azithromycin. J. Chemother. 18, 559–561. doi: 10.1179/joc.2006.18.5.559, PMID: 17127236

[ref33] Phin N. Parry-Ford F. Harrison T. Stagg H. R. Zhang N. Kumar K.. (2014). Epidemiology and clinical management of Legionnaires’ disease. Lancet Infect. Dis. 14, 1011–1021. doi: 10.1016/S1473-3099(14)70713-3 24970283

[ref34] Prussin A. J. Schwake D. O. Marr L. C. (2017). Ten questions concerning the aerosolization and transmission of Legionella in the built environment. Build. Environ. 123, 684–695. doi: 10.1016/j.buildenv.2017.06.024, PMID: 29104349 PMC5665586

[ref35] Qin T. Zhao D. Zhu L. Ren H. Li Y. Liu X.. (2022). *Legionella pneumophila* risk from cooling tower systems in China. Appl. Environ. Microbiol. 88:e0192121. doi: 10.1128/AEM.01921-21, PMID: 34818106 PMC8824207

[ref36] Samrakandi M. M. Cirillo S. L. Ridenour D. A. Bermudez L. E. Cirillo J. D. (2002). Genetic and phenotypic differences between *Legionella pneumophila* strains. J. Clin. Microbiol. 40, 1352–1362. doi: 10.1128/JCM.40.4.1352-1362.2002, PMID: 11923356 PMC140379

[ref37] Sawczyn-Domańska A. (2021). Detection of Legionella spp. and occurrence of virulence genes: lvh, rtxA and enhC in water samples from artificial water systems. Ann. Agric. Environ. Med. 28, 617–620. doi: 10.26444/aaem/143745 34969219

[ref38] Segal G. Russo J. J. Shuman H. A. (1999). Relationships between a new type IV secretion system and the icm/dot virulence system of *Legionella pneumophila*. Mol. Microbiol. 34, 799–809. doi: 10.1046/j.1365-2958.1999.01642.x, PMID: 10564519

[ref39] Sreenath K. Chaudhry R. Vinayaraj E. V. Dey A. B. Kabra S. K. Thakur B.. (2020). Distribution of virulence genes and sequence-based types among *Legionella pneumophila* isolated from the water systems of a tertiary care hospital in India. Front. Public Health 8:596463. doi: 10.3389/fpubh.2020.596463, PMID: 33330340 PMC7719716

[ref40] Vandewalle-Capo M. Massip C. Descours G. Charavit J. Chastang J. Billy P. A.. (2017). Minimum inhibitory concentration (MIC) distribution among wild-type strains of *Legionella pneumophila* identifies a subpopulation with reduced susceptibility to macrolides owing to efflux pump genes. Int. J. Antimicrob. Agents 50, 684–689. doi: 10.1016/j.ijantimicag.2017.08.001, PMID: 28782709

[ref41] WHO (2007). Legionella and the prevention of legionellosis: World Health Organization, Geneva, CH: WHO Press.

[ref42] Woodhead M. Blasi F. Ewig S. Garau J. Huchon G. Ieven M.. (2011). Guidelines for the management of adult lower respiratory tract infections--full version. Clin. Microbiol. Infect. 17 Suppl 6, E1–E59. doi: 10.1111/j.1469-0691.2011.03672.x, PMID: 21951385 PMC7128977

[ref43] Yu V. L. Plouffe J. F. Pastoris M. C. Stout J. E. Schousboe M. Widmer A.. (2002). Distribution of Legionella species and serogroups isolated by culture in patients with sporadic community-acquired legionellosis: an international collaborative survey. J. Infect. Dis. 186, 127–128. doi: 10.1086/341087, PMID: 12089674

